# Association Between Photodynamic Therapy Session Number and Psychosexual Well-Being in Vulvar Lichen Sclerosus: A Correlation Analysis

**DOI:** 10.3390/ph19091450

**Published:** 2026-09-13

**Authors:** Katarzyna Beutler-Rucińska, Alina Jankowska-Konsur, Danuta Nowicka

**Affiliations:** 1 University Centre of General Dermatology and Oncodermatology, Wroclaw Medical University, 50-556 Wroclaw, Poland; kasia.beutler@gmail.com (K.B.-R.); alina.jankowska-konsur@umw.edu.pl (A.J.-K.); 2 Division of Aesthetic Dermatology and Regenerative Medicine of the Skin, Wroclaw Medical University, 50-368 Wroclaw, Poland

**Keywords:** vulvar lichen sclerosus, photodynamic therapy, chronic inflammatory dermatosis, 5-aminolevulinic acid, female sexual functioning

## Abstract

**Background:** Vulvar lichen sclerosus (VLS) represents a chronic inflammatory dermatosis associated with significant symptoms, architectural changes of the vulva, and impaired quality of life (QoL). Photodynamic therapy (PDT) with 5-aminolevulinic acid (5-ALA) has emerged as a potential therapeutic option for patients with disease refractory to standard topical treatments. The aim was to investigate whether PDT is associated with QoL and sexual functioning outcomes. **Methods:** Patients with refractory VLS were treated with PDT using a 10% 5-ALA nanoemulsion applied to affected areas and covered with an aluminum foil occlusive dressing. Participants underwent between one and six PDT sessions, each consisting of 10 min of LED illumination (37 J/cm^2^, approximately 77 mW/cm^2^), delivered at 4–6-week intervals. Outcomes were assessed using the Dermatology Life Quality Index (DLQI; *n* = 37) and the Female Sexual Function Index (FSFI; *n* = 20). **Results:** A greater number of PDT sessions was associated with better quality of life (lower DLQI score; tau = −0.5831, adjusted *p* = 0.0002) and improved overall sexual function (higher FSFI score; tau = 0.5562, adjusted *p* = 0.0143). Analysis of individual FSFI domains showed significant positive correlations for desire (tau = 0.5125, adjusted *p* = 0.0166), arousal (tau = 0.4734, adjusted *p* = 0.0201), orgasm (tau = 0.4878, adjusted *p* = 0.0201), and satisfaction (tau = 0.5075, adjusted *p* = 0.0166), while lubrication (tau = 0.2019, adjusted *p* = 0.3960) and pain (tau = 0.0407, adjusted *p* = 0.8707) were not significantly associated with the number of PDT sessions. **Conclusions:** This study demonstrates an association between a higher number of PDT sessions and better quality-of-life and sexual-function outcomes in patients with refractory VLS. The findings support an individualized treatment approach guided by clinical response rather than fixed session limits. Future studies should focus on optimizing treatment protocols and identifying predictors of response.

## 1. Introduction

Vulvar lichen sclerosus (VLS) represents a chronic inflammatory dermatosis that mostly affects the anogenital area and is typified by gradual architectural alterations, white atrophic plaques, and epithelial thinning. The condition has a bimodal age distribution, with prepubertal girls and postmenopausal women experiencing the highest prevalence, while it can happen at any age [1,2]. In the overall female population, the estimated frequency varies between 0.6% and 1.7% [3]. Although the etiology of VLS remains incompletely understood, the information now available points to a complex pathophysiology that includes hormonal, immunological, and genetic components [1]. The condition is particularly common during hypoestrogenic phases, such as prepuberty and postmenopause, and is closely linked to autoimmune disorders [4]. A Th1-specific interferon-gamma (IFN-γ)-induced phenotype has been discovered by recent molecular research, and keratinocytes are crucial as generators of inflammatory alarmins and targets of inflammatory signaling [5]. Circulating autoantibodies against extracellular matrix proteins, oxidative stress, and abnormal tissue remodeling all contribute to the development of illness and may foster an environment that is favorable to malignant transformation [1].

Clinically, VLS manifests as distinctive white, thinned, atrophic plaques that are usually seen in the vulva and perianal area in a figure-eight pattern. Patients frequently suffer from burning, dyspareunia, dysuria, and intense itching. Significant functional impairment and a reduced quality of life are the results of severe instances when persistent scarring causes loss of vulvar architecture, including introital stenosis, clitoral phimosis, and regression of the labia minora [1,4]. Importantly, researchers report a high disease burden in patients with VLS and note that patients reported outcomes should be an integral part of treatment assessment [6,7,8]. The distinctive appearance of white, atrophic plaques in the anogenital area serves as the primary basis for the clinical diagnosis. However, in situations where there is aberrant presentation, treatment failure, or suspicion of cancer, vulvar biopsy is advised for histological confirmation. Histologically, VLS shows basal cell vacuolization, hyperkeratosis, epidermal atrophy, and a band-like lymphocytic infiltration in the upper dermis with collagen homogeneity underneath [9].

Long-term administration of ultrapotent topical corticosteroids, most frequently clobetasol propionate 0.05% ointment, is the gold-standard therapy for VLS. Topical calcineurin inhibitors (pimecrolimus, tacrolimus) are an alternative therapy; nevertheless, they are often saved for individuals who are intolerant to corticosteroids [1]. For individuals with corticosteroid-resistant or refractory conditions, photodynamic therapy (PDT) has been explored as an alternative or adjunctive treatment in patients with refractory VLS, but it is not currently endorsed as standard therapy in disease-specific guidelines [10]. PDT has been shown in several trials and systematic reviews to considerably reduce pruritus, improve clinical appearance, and improve quality of life in patients who have not responded to conventional therapy [11,12]. PDT works by applying a photosensitizer topically, usually 5-aminolevulinic acid (5-ALA), which is accumulated in the skin that is impacted. Red light, usually between 630 and 635 nm, causes 5-ALA to change into protoporphyrin IX, which produces reactive oxygen species (ROS) when oxygen is present. Clinical improvement results from the apoptosis and necrosis that these ROS cause in the keratinocytes, fibroblasts, and inflammatory cells that are present in the lesional tissue [11,12]. According to immunohistochemical research, PDT helps to alleviate symptoms and restore tissue architecture by decreasing local inflammation, increasing microvessel density (CD34+), and reducing lymphocytic infiltration (CD3+ T cells). The treatment also causes lesional cells to undergo apoptosis, which is linked to a decrease in pain and pruritus in particular [13,14]. Typically, the only reported side effects are temporary discomfort, erythema, and edema at the treatment location.

Overall, focused cytotoxicity and immunomodulation result in VLS clinical alleviation and histological improvement [12,14]. Current clinical guidelines do not specify a definitive recommended number of PDT sessions for VLS. The recently published EuroGuiDerm Guideline on Lichen Sclerosus (2024) does not include PDT among its primary recommendations, focusing instead on ultrapotent or potent topical corticosteroids as the gold standard of care, with topical calcineurin inhibitors as second-line treatment [15]. Nevertheless, PDT is receiving increasing attention from researchers and clinicians as a potential adjunctive option for refractory VLS. In this context, the Chinese expert consensus on the clinical application of ALA-based PDT in female lower genital tract diseases recognized the relevance of PDT in VLS and provided practical guidance for its clinical use, while also emphasizing that further evidence-based clinical studies are needed to update and expand current recommendations [16]. In line with these recommendations, we conducted the study with PDT to broaden existing knowledge.

The primary aim of this study was to investigate whether an association exists between the number of PDT sessions and clinical outcomes in patients with refractory VLS. Specifically, we sought to determine whether increasing treatment intensity is associated with progressive improvements in quality of life (assessed by DLQI) and sexual function (assessed by FSFI). This represents a novel contribution to existing literature, as previous studies have employed fixed treatment protocols without examining whether additional sessions beyond predetermined numbers confer incremental clinical benefit.

## 2. Results

**Figure 1:**
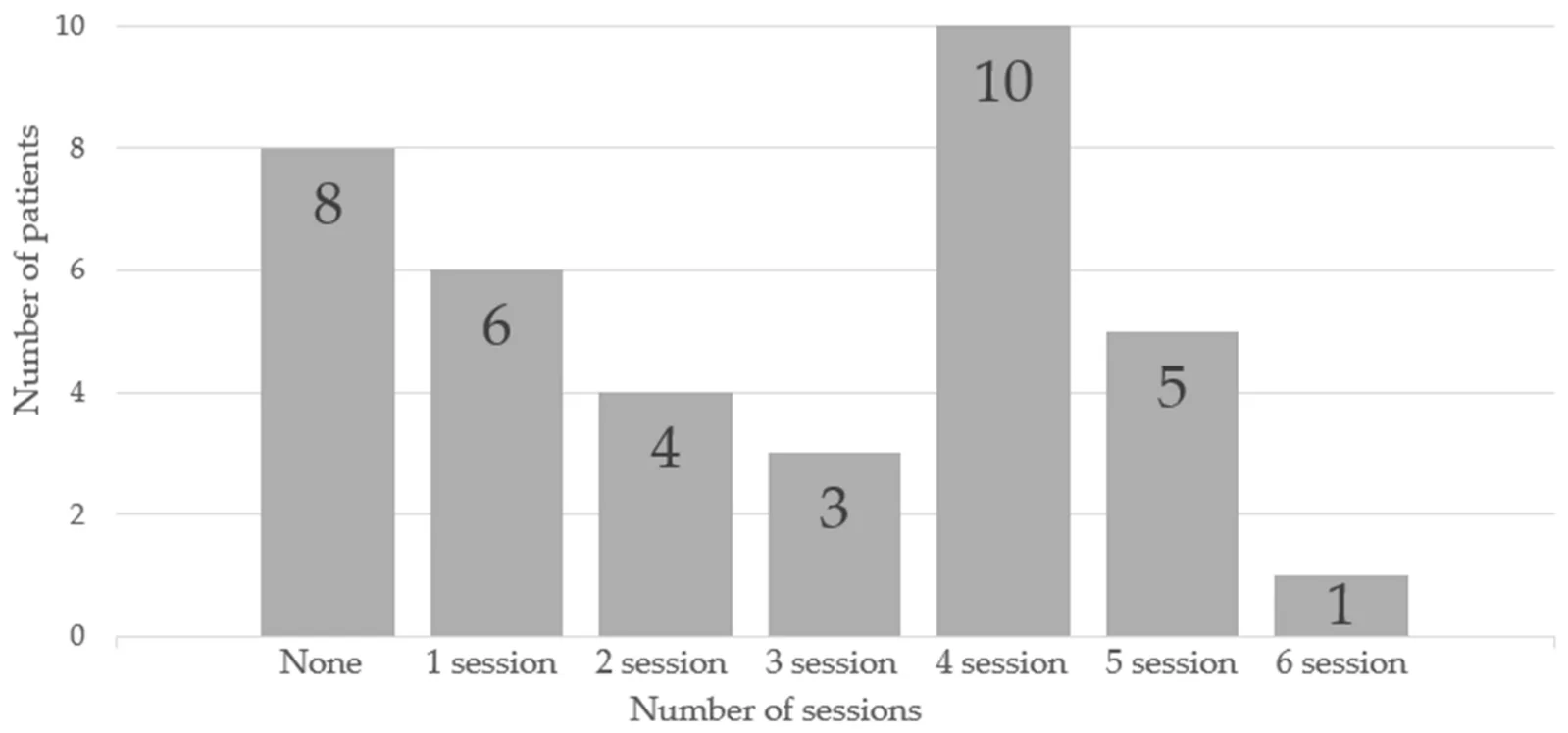
Patient distribution according to the number of PDT sessions.

The statistical evaluation proceeded through two distinct analytical stages determined by the applicability of the measurement instruments. Assessment of quality of life, based on the Dermatology Life Quality Index (DLQI), encompassed all 37 enrolled participants. Evaluation of sexual function based on the Female Sexual Function Index (FSFI), incorporating both the overall FSFI score and its component domains, encompassed 20 participants who confirmed current sexual activity. Of the 37 patients, 8 did not undergo PDT due to personal decision. Among the remaining patients, 6 received one PDT session, 4 received two sessions, 3 received three sessions, 10 received four sessions, 5 received five sessions, and 1 received six sessions (Figure 1). We compared patients who underwent PDT with those who did not receive treatment (Table 1). The groups differed significantly in DLQI score. Although FSFI scores were numerically more favorable in the treated group, the difference did not reach statistical significance.

This sample size for sexual function analysis, while limited, is consistent with the specialized nature of the study population (refractory VLS patients who are sexually active). Findings are displayed using both condensed presentation (correlation matrices) and comprehensive visualization incorporating distribution graphics and scatter diagrams to illustrate associations between examined variables.

The correlation analysis revealed statistically significant associations between the number of PDT sessions and the scores received for quality of life and sexual function (Table 2).

**Figure 2:**
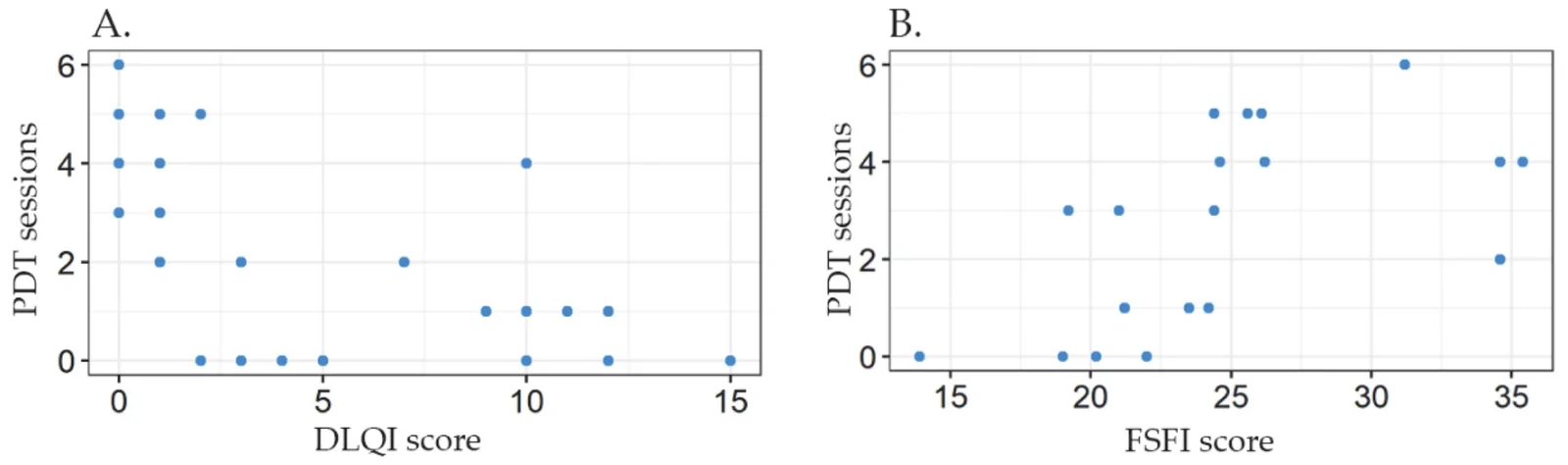
Correlation plots for DLQI (**A**) and FSFI (**B**), and the number of PDT sessions. Scatter plots display individual patient data points with fitted regression lines and 95% confidence intervals. The left panel (**A**) demonstrates the inverse relationship between DLQI scores and PDT session count, while the right panel (**B**) illustrates the positive association between FSFI scores and treatment intensity.

In the quality-of-life assessment, DLQI scores were strongly negatively correlated with the number of PDT sessions (tau = −0.583, adjusted *p* = 0.0002). This inverse relationship indicates that increasing treatment frequency was associated with decreasing DLQI values, reflecting improved dermatological quality of life, as lower DLQI scores denote lesser disease impact. Analysis of sexual function demonstrated a moderate positive correlation between overall FSFI scores and PDT session count (tau = 0.556, adjusted *p* = 0.014). Higher FSFI values correspond to better sexual function, suggesting that greater treatment intensity was associated with improved sexual outcomes. Correlation plots for DLQI and FSFI, and the number of PDT sessions, are presented in Figure 2.

Examination of individual FSFI domains revealed differential responsiveness to therapy:

- Domain I (Desire): Significant positive correlation (tau = 0.513, adjusted *p* = 0.017).
- Domain II (Arousal): Significant positive correlation (tau = 0.473, adjusted *p* = 0.020).
- Domain III (Lubrication): No significant association (tau = 0.202, adjusted *p* = 0.396).
- Domain IV (Orgasm): Significant positive correlation (tau = 0.488, adjusted *p* = 0.020).
- Domain V (Satisfaction): Significant positive correlation (tau = 0.507, adjusted *p* = 0.017).
- Domain VI (Pain): No significant association (tau = 0.041, adjusted *p* = 0.871).

**Figure 3:**
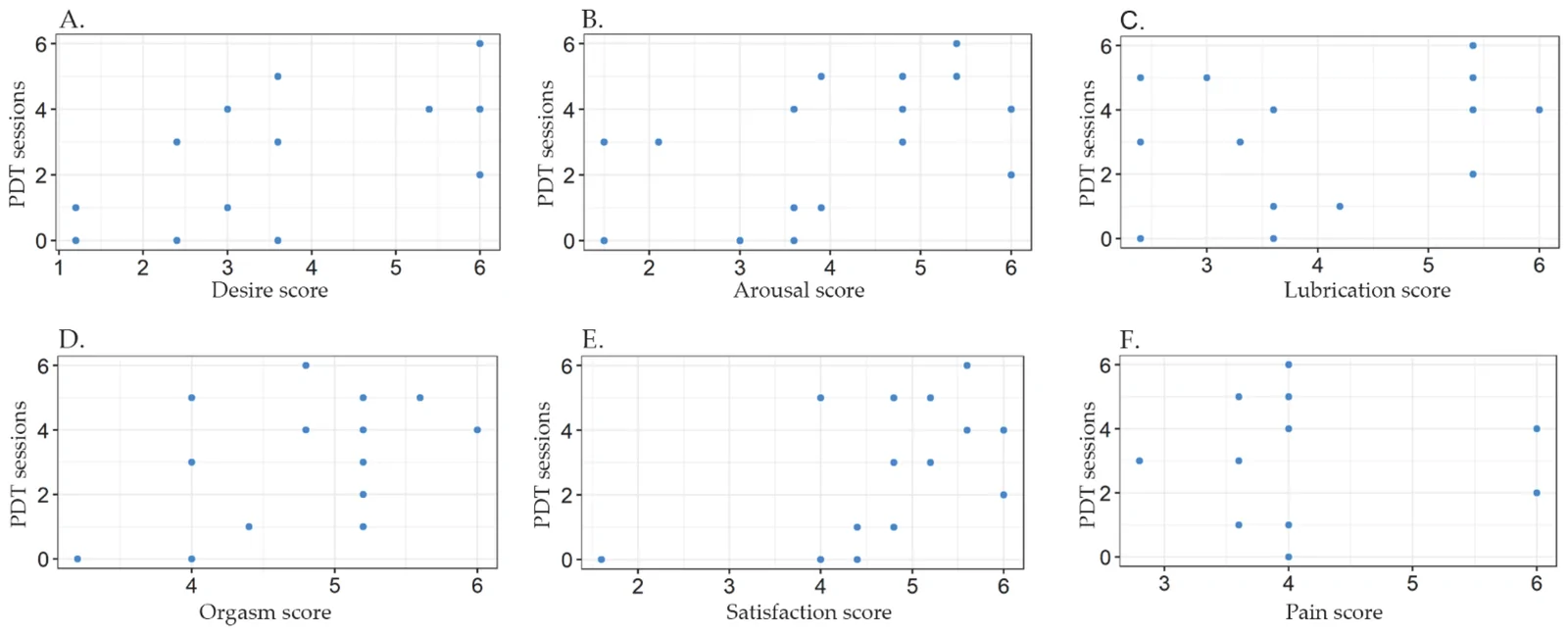
Correlation plots for the number of PDT sessions and scores of FSFI domains ((**A**) desire; (**B**) arousal; (**C**) lubrication; (**D**) orgasm; (**E**) satisfaction; (**F**) pain). Individual scatter plots with regression lines and 95% confidence intervals are presented for each of the six FSFI domains. Statistically significant correlations (adjusted *p* < 0.05) are indicated for desire, arousal, orgasm, and satisfaction domains.

These findings suggest that PDT exerts beneficial effects across most sexual function dimensions, with particularly notable improvements in desire, arousal, orgasm, and satisfaction. However, the lubrication and pain domains did not demonstrate statistically significant changes in relation to treatment frequency. Detailed results are presented in Figure 3.

## 3. Discussion

The present study shows an association between the number of PDT sessions and clinical outcomes in vulvar lichen sclerosus. The strong negative correlation between PDT session count and DLQI scores (tau = −0.583, adjusted *p* = 0.0002), alongside the moderate positive correlation with total FSFI scores (tau = 0.556, adjusted *p* = 0.014), demonstrates that increasing treatment intensity yields progressively greater improvements in both quality of life and sexual function. These findings have significant clinical implications, suggesting that therapeutic optimization may require individualized treatment duration rather than adherence to predetermined session numbers. Despite these encouraging results, PDT should be interpreted as an investigational or adjunctive therapeutic option in refractory VLS rather than as guideline-endorsed standard therapy. This distinction is clinically relevant, because the current disease-specific guideline framework continues to prioritize ultrapotent or potent topical corticosteroids, with topical calcineurin inhibitors reserved as second-line treatment, whereas PDT requires further controlled evidence before it can be incorporated into routine guideline-based management.

### 3.1. Comparison with Fixed-Protocol Studies

The associations with the number of PDT sessions observed in this study represent a novel contribution to the existing literature [11,17]. While previous studies have established PDT efficacy using fixed treatment protocols, the correlation analysis uniquely demonstrates that cumulative treatment sessions produce incremental clinical benefits. Zhang et al. employed a standardized protocol of three 5-ALA-PDT cycles in 30 patients with refractory VLS, reporting complete disappearance of pruritus in 27 cases (90%) and significant quality of life improvement (*p* < 0.001) [12]. Similarly, Shi et al. administered four PDT sessions at two-week intervals, achieving a complete response rate of 70% (14/20 patients), which was double that observed with clobetasol propionate (35%, *p* < 0.05) [18]. However, neither study examined whether additional sessions beyond their fixed protocols would confer further benefit. The present findings extend these observations by demonstrating that patients receiving more sessions exhibit superior outcomes across multiple domains. This aligns with early observations by Hillemanns et al. in their pioneering pilot study, where some patients required multiple PDT cycles—two women underwent two cycles, and one underwent three cycles—with a prolonged therapeutic effect averaging 6.1 months [19]. The concept of cumulative photodynamic tissue remodeling is further supported by Bizoń et al., who, in a large cohort of 182 women, reported quality of life improvement in 91.3% of patients, with objective vulvoscopic assessment confirming reduction of vulvar changes by approximately 21% [20].

### 3.2. Mechanisms Underlying the Association with the Number of Sessions

The progressive improvement observed with increasing PDT sessions may be explained by the cumulative effects of photodynamic therapy on tissue remodeling [21]. Karrer et al. demonstrated that 5-ALA-PDT induces significant, time-dependent increases in matrix metalloproteinase-1 (up to 2.4-fold after 48 h) and matrix metalloproteinase-3 (up to 4.3-fold after 48 h) protein levels, while simultaneously reducing collagen type I mRNA expression [22]. This dual mechanism—induction of collagen-degrading enzymes together with the reduction of collagen production—may be responsible for the anti-sclerotic effects observed clinically and could explain why repeated treatments produce cumulative tissue normalization. Furthermore, Jang et al. (2013) demonstrated that PDT induces prolonged activation of extracellular signal-regulated kinase (ERK) in dermal fibroblasts, leading to increased fibroblast proliferation and collagen type Iα expression [23]. Additionally, Karrer et al. demonstrated that PDT triggers MMP production in dermal fibroblasts not only directly but also through an indirect paracrine loop mediated by soluble factors released by epidermal keratinocytes, particularly interleukin-1α [24]. This complex interplay between keratinocytes and fibroblasts may require multiple treatment sessions to achieve optimal tissue remodeling, further explaining why greater treatment intensity correlates with superior clinical outcomes. Olejek et al. provided immunohistochemical evidence supporting PDT’s mechanism of action in VLS, demonstrating statistically significant increases in microvessel density (anti-CD34 staining, *p* < 0.05) and decreases in lymphocytic infiltration (CD3 expression, *p* < 0.05) following six PDT courses [14]. These findings suggest that PDT exerts both pro-angiogenic and anti-inflammatory effects, which may accumulate with repeated treatments.

### 3.3. Influence of VLS-Associated Histopathological Changes on 5-ALA Penetration

The characteristic histopathological features of VLS—hyperkeratosis, epidermal atrophy, and dense dermal sclerosis—have important implications for 5-ALA transdermal kinetics and therapeutic efficacy. Understanding these interactions is essential for optimizing PDT protocols in this patient population. Hyperkeratosis represents a significant barrier to ALA penetration, as the stratum corneum constitutes the primary obstacle for hydrophilic ALA molecules. Thickened keratinized layers can reduce drug uptake and subsequent PpIX accumulation [25,26]. However, in the present study, this barrier was addressed through two specific strategies. First, the use of a nanoemulsion formulation of 5-ALA has been demonstrated to achieve significantly deeper penetration compared to conventional formulations—studies with nanoemulsion-based ALA showed PpIX fluorescence at depths of 97.2 ± 5.7 μm versus 42.0 ± 4.2 μm for standard formulations [27]. Second, the application of an aluminum foil occlusive dressing hydrates the stratum corneum, disrupts its lipid organization, and enhances ALA permeation [28]. Paradoxically, the epidermal atrophy characteristic of VLS may actually facilitate rather than hinder 5-ALA penetration. A thinned epidermis presents a reduced diffusion barrier, potentially allowing more rapid and deeper penetration of ALA molecules. This is consistent with the observation that ALA penetrates more readily when the epidermal barrier is impaired [29]. Furthermore, ALA has a low molecular weight that enables it to penetrate even intact stratum corneum [30], and a thinned atrophic epidermis would be expected to offer even less resistance to drug diffusion. Dense dermal sclerosis, characterized by homogenized collagen and reduced vascularity, could theoretically impede deeper drug distribution and reduce the availability of cellular targets. However, several considerations mitigate this concern. The primary therapeutic target of ALA-PDT in VLS is the inflammatory infiltrate and epidermal/subepidermal compartment, not the deep dermis. The relevant penetration depth for therapeutic effect is within the range achievable by nanoemulsion formulations [31]. Importantly, ALA-PDT has been shown to exert direct anti-sclerotic effects by inducing matrix metalloproteinases (MMP-1 and MMP-3) in a singlet oxygen-dependent manner while simultaneously reducing collagen type I mRNA expression in both normal and scleroderma fibroblasts [22]. This suggests that even limited ALA penetration into sclerotic tissue can initiate a therapeutic cascade that remodels the pathological extracellular matrix. Studies on hypertrophic scars—another condition characterized by dense collagen deposition—have demonstrated that nanoemulsion and nanoethosome formulations of ALA can overcome the compact dermal barrier and deliver ALA into deep fibrotic lesions [32]. The efficacy of treatment observed in the present study and in previous investigations confirms that sufficient ALA penetration occurs in VLS-affected tissue to achieve therapeutic effects. Multiple studies have demonstrated clinical and histopathological improvement following ALA-PDT in VLS, including reduction of the inflammatory infiltrate and induction of apoptosis [22,33]. Nevertheless, inter-patient variability in the degree of hyperkeratosis, atrophy, and sclerosis may contribute to heterogeneous treatment responses, which is consistent with our recommendation for an individualized, response-guided treatment approach.

### 3.4. Sexual Function Outcomes and Domain-Specific Responses

The differential response across FSFI domains provides important insights into PDT’s therapeutic action. Significant improvements in desire (tau = 0.513), arousal (tau = 0.473), orgasm (tau = 0.488), and satisfaction (tau = 0.507) domains suggest that repeated PDT sessions progressively restore vulvar tissue integrity and reduce inflammatory burden, thereby enhancing sexual responsiveness. These findings are particularly relevant given the high prevalence of sexual dysfunction in VLS patients. Pope et al., in a systematic review and meta-analysis, reported that nearly 60% of women with lichen sclerosus suffer from sexual dysfunction (95% CI: 48–70%), with dyspareunia being the most commonly reported type [34]. The absence of significant correlation in lubrication (tau = 0.202, adjusted *p* = 0.396) and pain (tau = 0.041, adjusted *p* = 0.871) domains warrants careful consideration. Caspersen et al. in a mixed-methods study of 172 women with VLS demonstrated that sexual dysfunction has a complex biopsychosocial nature, with 68% of sexually active women meeting international criteria for sexual dysfunction [35]. The authors identified that pain during intercourse may be perpetuated by psychological mechanisms even after resolution of inflammatory changes, which could explain the limited response in the pain domain despite multiple PDT sessions. Furthermore, Gerkowicz et al. in their systematic review emphasized that VLS patients exhibit focal atrophy and destructive scarring that may be irreversible despite effective anti-inflammatory therapy [11]. The lubrication domain may be particularly resistant to improvement due to systemic hormonal factors in postmenopausal women—who constitute the majority of VLS patients—that PDT cannot address directly.

### 3.5. Comparison with Topical Corticosteroid Therapy

The association between the number of PDT sessions and clinical response observed in this study has important implications when comparing PDT with standard topical corticosteroid therapy. According to ACOG Practice Bulletin, medium-potency or high-potency topical corticosteroid ointment is recommended as first-line treatment for lichen sclerosus, with long-term individualized therapy recommended to maintain skin normality and prevent scarring [36]. However, Kohn et al., in a retrospective cohort study of 333 women, demonstrated that only 66% of patients used steroids exactly as prescribed, with adherence significantly affecting outcomes [37]. Importantly, 42% of women were sexually inactive due to pain at intake, and only 37% of these became sexually active after steroid treatment. In contrast, Shi et al. demonstrated that one month after completing PDT, only one patient relapsed compared to all seven responders in the clobetasol propionate group [18]. This striking difference in recurrence rates suggests that cumulative PDT sessions may produce more durable tissue changes than topical corticosteroids, potentially through the matrix remodeling mechanisms described above.

### 3.6. Comparison with Alternative Light-Based Therapies

The present findings should be contextualized within the broader landscape of light-based therapies for VLS. Zivanovic et al. in a randomized controlled trial comparing Nd:YAG/Er:YAG dual laser therapy with topical corticosteroids demonstrated significantly greater reduction in clinical LS score in the laser group (−2.34 ± 1.20 vs. −0.95 ± 0.90, *p* < 0.001), with higher patient satisfaction (*p* = 0.035) [38]. Notably, this study employed four laser treatments at 0, 1, 2, and 4 months, supporting the concept that repeated light-based interventions yield superior outcomes. Similarly, Pagano et al. evaluated fractional microablative CO_2_ laser in 40 women with VLS resistant to long-term topical corticosteroids, demonstrating significant improvement in vulvar itching (*p* < 0.001), vulvar dryness (*p* < 0.001), superficial dyspareunia (*p* < 0.001), and sensitivity during intercourse (*p* < 0.001) after two treatment cycles [39]. Campolmi et al. reported the successful use of fractional microablative CO_2_ laser in a 69-year-old man with treatment-refractory penile LS who underwent five monthly sessions. Symptom improvement and increased skin elasticity were observed after the first session, while complete clinical remission, restoration of sexual activity, and no relapse were reported 14 weeks after the final treatment; however, these findings were based on a single case [40]. Wei et al., in a systematic review of seven RCTs (332 patients), concluded that laser therapy improves symptoms, signs, quality of life, and histological outcomes in VLS, with three studies reporting greater symptom/sign improvement than topical corticosteroids (*p* < 0.05) [41]. These findings across multiple light-based modalities consistently support the principle that repeated treatments produce superior outcomes.

### 3.7. Clinical Implications for Treatment Planning

The correlation analysis suggests that PDT may be continued based on individual clinical response rather than discontinued after a fixed number of sessions. However, the optimal timing for achieving a satisfactory clinical response should be individualized and requires further study. This individualized approach is supported by the observation that patients receiving more sessions demonstrated progressively better quality of life and sexual function outcomes. Gerkowicz et al., in their systematic review of 20 studies, emphasized the necessity of implementing appropriate therapy at the earliest possible disease stage to avoid serious complications, including neoplastic transformation [11]. Given that VLS carries an increased risk of malignant transformation, the cumulative tissue-normalizing effects of multiple PDT sessions may have implications beyond symptom relief. The low recurrence rate observed with PDT compared to topical corticosteroids further supports extended treatment protocols. Zawislak et al., using a novel bioadhesive 5-ALA patch system, reported significant symptomatic relief in six of ten patients after 17 cycles of PDT, with statistically significant induction of apoptosis observed histopathologically [13]. This suggests that multiple treatment cycles may be necessary to achieve optimal cellular and tissue remodeling. The pharmacokinetic considerations discussed above—particularly the ability of nanoemulsion formulations to overcome hyperkeratotic barriers and the paradoxical facilitation of drug penetration by epidermal atrophy—further support the rationale for repeated treatment sessions to achieve cumulative therapeutic effects in tissues with variable histopathological features.

### 3.8. Study Limitations and Strengths

Several limitations should be acknowledged. The relatively small sample size, particularly for sexual function analysis (*n* = 20), may limit detection of weaker correlations and reduce generalizability. For this reason, the study group was not further stratified by age or menopausal status. However, this sample size is consistent with the specialized nature of the study population—patients with refractory VLS who are sexually active represent a subset of an already uncommon clinical condition. Furthermore, the statistical approach employed (Kendall’s tau correlation) is specifically designed for smaller samples and demonstrated adequate power to detect the moderate-to-strong correlations observed.

The correlation between the number of PDT sessions and DLQI was calculated in the entire enrolled cohort, including patients who declined treatment and did not receive any sessions. Because treatment initiation was not randomized and untreated participants had worse DLQI scores, the observed association may partly reflect baseline differences and self-selection rather than an effect of increasing treatment exposure. The small treated sample and uneven distribution of patients across session categories also limit the reliability of subgroup or treated-only analyses.

The absence of a control group precludes direct comparison with other therapeutic modalities or placebo effects. Moreover, the untreated patients were non-randomized decliners, and their inclusion may have influenced the observed correlations. While this represents an inherent limitation of the observational design, we note that the primary aim of this study was to examine the relationship between the number of PDT sessions and clinical outcomes rather than to compare PDT efficacy against other treatments. However, we also note that the number of PDT sessions may also reflect clinical course, treatment indication, adherence, or perceived benefit rather than an independent therapeutic dose.

Furthermore, the observational design cannot establish causality—patients receiving more sessions may have had more severe disease requiring extended treatment, or conversely, may have been more adherent due to perceived benefit. The lack of long-term follow-up data prevents assessment of the durability of the assessed relationship. Additionally, inter-patient variability in histopathological features (degree of hyperkeratosis, epidermal atrophy, and dermal sclerosis) was not systematically assessed, which may have contributed to heterogeneity in treatment response. Strengths of this study include the use of validated assessment instruments (DLQI, FSFI), which are internationally recognized gold-standard tools with established psychometric properties for measuring quality of life and sexual function outcomes. The DLQI has been extensively validated in dermatological conditions and provides reliable, reproducible measurements of disease impact on daily life. Similarly, the FSFI demonstrates excellent reliability and sensitivity to treatment-related changes across diverse populations and clinical conditions. While these instruments rely on patient self-report, they capture the subjective experience of symptoms and functional impairment that is most clinically meaningful to patients—aspects that objective clinical assessments alone cannot fully capture. Detailed domain-level analysis of sexual function and the novel examination of the number of sessions during PDT for VLS represent additional methodological strengths. The application of a nanoemulsion 5-ALA formulation with occlusive dressing represents an optimized drug delivery strategy designed to overcome the penetration barriers inherent to VLS-affected tissue. The application of appropriate statistical methods, including Kendall’s tau correlation and FDR correction for multiple comparisons, ensures robust interpretation of results. The findings provide a foundation for evidence-based treatment protocols emphasizing continued therapy until clinical plateau rather than arbitrary session limits.

### 3.9. Future Directions

Future randomized controlled trials with predetermined session numbers and extended follow-up periods would help clarify the optimal treatment intensity and durability of response. Such trials should incorporate both patient-reported outcomes (DLQI, FSFI) and objective clinical assessments to provide complementary perspectives on treatment efficacy. Studies comparing different PDT protocols (varying session numbers, intervals, and light doses) would bring more insight into this type of treatment. Additionally, the investigation of biomarkers predicting response to PDT could enable personalized treatment planning. Future studies should also investigate the correlation between baseline histopathological features (degree of hyperkeratosis, epidermal thickness, and dermal sclerosis) and treatment response, which could help identify patients most likely to benefit from PDT and inform the development of personalized treatment protocols. Pharmacokinetic studies using confocal microscopy or fluorescence imaging to assess PpIX accumulation in VLS tissue with varying histopathological characteristics would provide valuable insights into optimizing drug delivery. Future studies should implement systematic quantitative documentation of adverse events, including standardized severity scales (mild/moderate/severe) for pain, erythema, and edema, to strengthen safety profiling and enable more robust tolerability comparisons across treatment protocols. The integration of PDT with other therapeutic modalities, such as topical corticosteroids or calcineurin inhibitors, warrants investigation to determine whether combination approaches might optimize outcomes while minimizing treatment burden. Finally, long-term studies assessing the impact of cumulative PDT sessions on malignant transformation risk would address an important clinical concern in VLS management.

## 4. Materials and Methods

### 4.1. Attendees

This research was performed from June 2025 through February 2026 at the University Center for General and Oncological Dermatology of the Medical University of Wrocław.

Patient enrollment involved 40 individuals with vulvar lichen sclerosus scheduled for PDT. All included patients had clinically confirmed VLS with persistent symptoms despite previous topical treatment with glucocorticosteroids and calcineurin inhibitors, indicating a refractory study population. The clinical stage of VLS, symptom severity scores, and lesion area were not systematically assessed at baseline. Following the withdrawal of three participants, the final analysis included 37 patients. The cohort consisted of women ranging in age from 25 to 87 years (mean 61.08 ± 14.4). Participants were required to be at least 18 years old with vulvar lichen sclerosus verified by clinical assessment or histopathological analysis. Patients were excluded if they had photodermatoses, were pregnant or lactating, presented with active infections in the treatment area, or were taking photosensitizing drugs. Among the 37 enrolled patients, histopathological confirmation was obtained in 17 cases (46%), while 20 cases (54%) were diagnosed clinically by dermatologists. Following inadequate response to conventional therapies—including topical clobetasol propionate (0.05%) and topical tacrolimus (0.1%)—participants were selected for photodynamic therapy and initiated treatment. Participants answered the DLQI and FSFI questionnaires after completion of the last PDT session. All 37 patients provided DLQI responses, whereas three declined FSFI completion without explanation, and 14 reported sexual inactivity. The restriction of FSFI analysis to sexually active participants (*n* = 20) was methodologically necessary, as the FSFI instrument generates systematically low scores in sexually inactive individuals that reflect absence of activity rather than dysfunction, which would confound interpretation of treatment effects. PDT represents an established treatment modality for vulvar lichen sclerosus at the University Center for General and Oncological Dermatology. The Bioethics Committee of the Piastów Śląskich Medical University in Wrocław determined that this study did not require formal ethics committee approval (certificate no. 474/2025).

### 4.2. Protocol for Treatment

#### 4.2.1. Preparation and Photosensitizer Application

Before initiating therapy, the vulvar lesions underwent meticulous cleansing and antiseptic preparation. A photosensitizing agent [Ameluz^®^, Leverkusen, Germany, formulated with 5-aminolevulinic acid hydrochloride (5-ALA HCl) 78 mg/g], equivalent to 10% pure 5-ALA, was applied uniformly across the affected tissue in an approximately 1 mm thick layer using a sterile applicator. Subsequently, an occlusive barrier (aluminum foil) was positioned over the treatment site to block light exposure and maximize photosensitizer uptake. The incubation phase extended for three hours.

#### 4.2.2. Pain Control Strategy

To minimize treatment-associated pain, oral analgesics were given one hour before illumination. Participants received either 1 g paracetamol or a combination formulation containing tramadol (37.5 mg) and paracetamol (325 mg), selected according to individual pain sensitivity and clinical assessment.

#### 4.2.3. Illumination Phase

After incubation completion, the protective covering was removed and excess photosensitizer was thoroughly cleaned away. To safeguard against urinary tract irritation, the urethral opening was shielded before light delivery. Light exposure was delivered via the BF-RhodoLED apparatus (Biofrontera AG, Leverkusen, Germany), a certified medical instrument designed for PDT applications. Target tissues received red light at a 635 nm central wavelength. A standardized therapeutic protocol was employed, administering a total fluence of 37 J/cm^2^ at a maximum irradiance of 77 mW/cm^2^ ± 15%. Although the lamp’s nominal irradiance was approximately 77 mW/cm^2^, in our treatment protocol, a greater distance between the light source and the irradiated area was maintained, resulting in a lower effective irradiance at the skin surface and a delivered fluence of 37 J/cm^2^ over 10 min. The device’s built-in cooling mechanism operated continuously during illumination to reduce thermal discomfort and improve patient comfort.

#### 4.2.4. Post-Treatment Management and Follow-Up

Immediately after light exposure, a cooling emollient or regenerative topical agent was administered to the treated region. Participants received instructions for gentle intimate hygiene using non-aggressive cleansing products. Treatment courses ranged from 1 to 6 sessions per individual.

#### 4.2.5. Adverse Events and Tolerability

Common side effects included temporary pain during illumination, along with post-treatment swelling and redness, which generally subsided within days. While adverse events were tracked for safety surveillance, systematic quantitative documentation was not implemented in this investigation.

#### 4.2.6. Treatment Schedule

**Figure 4:**
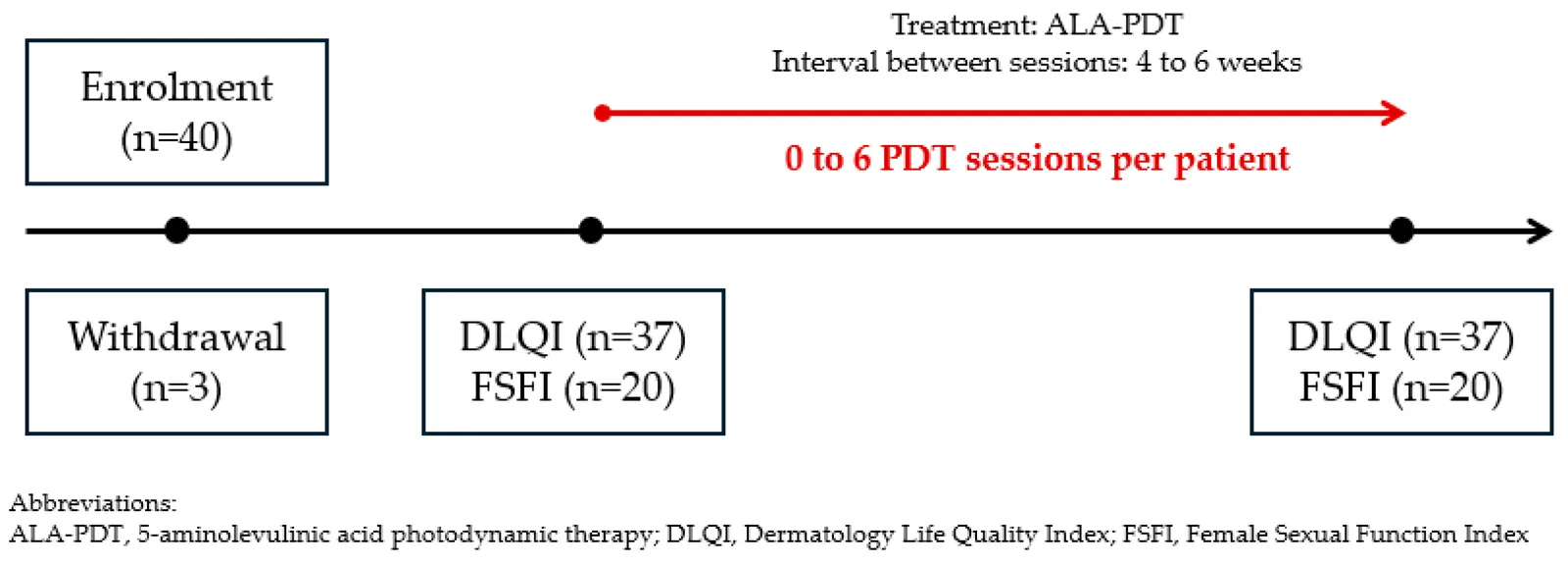
Study design.

Sessions were scheduled for 4–6-week intervals. The number of PDT treatments per participant ranged from 0 to 6, determined by individual therapeutic response and protocol compliance. The decision to continue or stop PDT was based on clinical assessment by the treating specialist, together with patient-reported improvement or persistence of symptoms. Additional sessions were offered when clinical signs or symptoms persisted and the patient agreed to continue treatment, whereas treatment was stopped when satisfactory clinical and subjective improvement was achieved. This variability in treatment frequency enabled correlation analysis between procedural intensity and improvements in quality of life and sexual function outcomes. The study protocol is presented in Figure 4.

### 4.3. Tools

For the analysis of PDT effectiveness and the relationship between the number of treatment sessions performed, two validated assessment instruments were employed: DLQI and FSFI.

DLQI is a widely utilized dermatology-specific questionnaire designed to evaluate the impact of skin conditions on patients’ quality of life. This instrument comprises 10 questions addressing various domains, including symptoms and feelings, daily activities, leisure, work and school performance, personal relationships, and treatment burden. Each item is scored on a 4-point scale (0–3), yielding a total score ranging from 0 to 30. Higher scores indicate greater impairment in quality of life, with 0–1 representing no effect, 2–5 small effect, 6–10 moderate effect, 11–20 very large effect, and 21–30 extremely large effect on the patient’s life. The DLQI has demonstrated robust psychometric properties and is considered the gold standard for assessing quality of life in dermatological conditions [42].

FSFI is a validated, multidimensional self-report instrument for evaluating female sexual function. This questionnaire consists of 19 items organized into six domains: desire (2 items), arousal (4 items), lubrication (4 items), orgasm (3 items), satisfaction (3 items), and pain (3 items). Responses are scored on a scale from 0 or 1 to 5, with domain scores calculated by summing individual item scores and multiplying by a domain-specific factor. The total FSFI score ranges from 2 to 36, with higher scores reflecting better sexual function. A total score below 26.55 is commonly used as a threshold for identifying female sexual dysfunction. The FSFI has been extensively validated across diverse populations and clinical conditions, demonstrating excellent reliability and sensitivity to treatment-related changes [43,44].

### 4.4. Data Assessment

Statistical analysis was performed in two separate stages, reflecting the distinct nature of the measurement tools and participant characteristics. The evaluation of dermatological impact on overall quality of life (assessed with DLQI) encompassed all enrolled participants (*n* = 37). In contrast, sexual function analysis utilizing the FSFI instrument was limited to 20 patients who confirmed sexual activity within the four-week period prior to assessment. This exclusion of sexually inactive participants was necessary to avoid systematic distortion in FSFI interpretation, as the absence of activity generates “0” scores that may falsely suggest clinical impairment rather than reflecting true dysfunction.

Groups were compared using the Mann–Whitney test due to the non-normal distribution of the data. Considering the ordinal structure of Likert-based responses and sample dimensions, Kendall’s tau (tau) rank correlation coefficient was selected to examine associations between variable pairs. This statistical approach was chosen for its resistance to extreme values and superior accuracy in smaller datasets relative to Spearman’s method. The nature of relationships was indicated by the tau coefficient’s polarity (positive values denoting direct associations, negative values indicating inverse relationships).

The primary significance threshold was established at *p* = 0.05. To account for multiple comparisons and reduce Type I error probability, the Benjamini–Hochberg False Discovery Rate (FDR) correction was implemented on the obtained *p*-values. This strategy provided stringent control over false-positive proportions. Findings are displayed in a comprehensive correlation matrix, with notable relationships additionally illustrated through distribution graphics and scatter diagrams. All computational procedures were executed using R software version 4.5.2.

## 5. Conclusions

This study suggests that, in patients with refractory VLS, a higher number of PDT sessions may be associated with greater improvements in quality of life and sexual function. These results position PDT as a potentially effective intervention for patients suffering from this chronic condition that profoundly impacts both dermatological health and intimate function. The findings support an individualized approach to treatment duration, with continuation of therapy guided by ongoing clinical response rather than adherence to arbitrary session limits. Due to the small sample size, the results should be interpreted with caution, given the exploratory and hypothesis-generating nature of the analysis. Future research should focus on optimizing treatment protocols and identifying patient characteristics that predict response to extended PDT courses.

## Figures and Tables

**Table 1:** Comparison between patients with and without treatment with PDT.

Variable	Without PDT	With PDT	*p*-Value
Age	65.00 ± 19.74	60.00 ± 12.78	0.3181
DLQI	8.25 ± 5.39	3.59 ± 4.25	0.0049
FSFI	15.90 ± 7.10	16.10 ± 12.07	0.6785

DLQI, Dermatology Life Quality Index; FSFI, Female Sexual Function Index; PDT, photodynamic therapy.

**Table 2:** Correlation table between DLQI score, FSFI total score and FSFI domains versus the number of PDT sessions.

Variables	Tau	*p*-Value	Data	Adjusted *p*-Value *
DLQI vs. PDT	−0.5831	<0.0001	Entire population	0.0002
FSFI vs. PDT	0.5562	0.0012	FSFI population	0.0143
Desire vs. PDT	0.5125	0.0049	FSFI population	0.0166
Arousal vs. PDT	0.4734	0.0075	FSFI population	0.0201
Lubrication vs. PDT	0.2019	0.2640	FSFI population	0.3960
Orgasm vs. PDT	0.4878	0.0068	FSFI population	0.0201
Satisfaction vs. PDT	0.5075	0.0044	FSFI population	0.0166
Pain vs. PDT	0.0407	0.8298	FSFI population	0.8707

DLQI, Dermatology Life Quality Index; FSFI, Female Sexual Function Index; PDT, photodynamic therapy. * Adjusted *p*-value with FDR correction for multiple comparisons.

## Data Availability

The original contributions presented in this study are included in the article. Further inquiries can be directed to the corresponding author.

## Abbreviations

The following abbreviations are used in this manuscript:

5-ALA	5-Aminolevulinic acid
DLQI	Dermatology Life Quality Index
FDR	False Discovery Rate
FSFI	Female Sexual Function Index
IFN-γ	Interferon-gamma
PDT	Photodynamic therapy
ROS	Reactive oxygen species
VLS	Vulvar lichen sclerosus
